# Potential use of gold-silver core-shell nanoparticles derived from *Garcinia mangostana* peel for anticancer compound, protocatechuic acid delivery

**DOI:** 10.3389/fmolb.2022.997471

**Published:** 2022-10-11

**Authors:** Kar Xin Lee, Kamyar Shameli, Yuki Nagao, Yen Pin Yew, Sin-Yeang Teow, Hassan Moeini

**Affiliations:** ^1^ Malaysia-Japan International Institute of Technology (MJIIT), Universiti Teknologi Malaysia, Kuala Lumpur, Malaysia; ^2^ School of Materials Science, Japan Advanced Institute of Science and Technology (JAIST), Nomi, Japan; ^3^ Department of Medical Sciences, School of Medical and Life Sciences (SMLS), Sunway University, Jalan Universiti, Bandar Sunway, Selangor Darul Ehsan, Malaysia; ^4^ School of Medicine, Institute of Virology, Technical University of Munich, Munich, Germany

**Keywords:** colorectal cancer, green synthesis, core-shell nanoparticles, gold-silver, protocatechuic acid, drug delivery, HCT116, CCD112

## Abstract

Colorectal cancer is one of the most killing cancers and this has become a global problem. Current treatment and anticancer drugs cannot specifically target the cancerous cells, thus causing toxicity towards surrounding non-cancer cells. Hence, there is an urgent need to discover a more target-specific therapeutic agent to overcome this problem. Core-shell nanoparticles have emerged as good candidate for anticancer treatment. This study aimed to synthesize core-shell nanoparticles *via* green method which utilised crude peels extract of *Garcinia mangostana* as reducing and stabilising agents for drug delivery. Gold-silver core-shell nanoparticles (Au-AgNPs) were synthesized through seed germination process in which gold nanoparticles acted as the seed. A complete coating was observed through transmission electron microscopy (TEM) when the ratio of AuNPs and AgNPs was 1:9. The size of Au-AgNPs was 38.22 ± 8.41 nm and was mostly spherical in shape. Plant-based drug, protocatechuic acid (PCA) was loaded on the Au-AgNPs to investigate their anticancer activity. In HCT116 colon cancer cells, PCA-loaded Au-AgNPs (IC_50_ = 10.78 μg/ml) showed higher inhibitory action than the free PCA (IC_50_= 148.09 μg/ml) and Au-AgNPs alone (IC_50_= 24.36 μg/ml). Up to 80% inhibition of HCT116 cells was observed after the treatment of PCA-loaded Au-AgNPs at 15.63 μg/ml. The PCA-loaded Au-AgNPs also showed a better selectivity towards HCT116 compared to CCD112 colon normal cells when tested at the same concentrations. These findings suggest that Au-AgNPs system can be used as a potent nanocarrier to combat cancerous cells by offering additional anticancer properties to the loaded drug.

## Highlights

• Through the green bio-synthesis method the gold-silver core-shell nanoparticles (Au-Ag NPs) synthesised by Garcinia mangostana crude peels extract as a reducing and stabilising agents.
• Structural, morphological, and compositional properties were investigated by the physicochemical characterization method.
• Protocatechuic acid (PCA) as a plant-based drug has been loaded on Au-Ag NPs to investigate their anticancer activity against colon cancer cells.
• The nanodrug showed a better selectivity towards colon cancer cell (HCT116) compared to colon normal cells (CCD112) when tested at the same concentrations.

## Introduction

According to the Malaysia statistics (year 2012–2016), breast, colorectal, and lung cancers are among the most common human cancers across all ethnicity (Azizah et al., 2016). Colorectal cancer (CRC) emerges as a deadly cancer among others (Xu et al., 2019). Patients diagnosed with CRC often have to undergo surgery, chemo- and/or radio-therapy. Chemotherapy is frequently used to treat CRC especially at the late stages (Shen et al., 2019), other newer therapies are monoclonal antibody and immunotherapy. However, these treatments often accompany with undesired toxicities and side effects to human body (Nyst et al., 2009). Healthy tissues surrounding the tumours tend to take up the chemotherapeutic drugs as well due to the poor drug selectivity and specificity (Benoist and Bernard 2009). Therefore, the focus of researches nowadays is to improve the efficiency as well as the specificity of these drugs. One of the approaches is to use a novel and non-toxic nanoparticles (NPs)-based drug delivery system which is superior in improving drug bio-distribution, selectivity, dosage efficiency, and therapeutic indices (Cho et al., 2008).

Nanotechnology has been an exciting field by introducing a novel drug delivery system. Nanoparticles can act as a highly versatile platform for the combination of therapeutic agents and tracking agents. When specific features are developed such as size, shape, hydrophobicity, surface properties, and other physicochemical characteristics, NPs of interest could specifically target diseased cells rather than offering non-target-specific delivery (Bendale, Bendale, and Paul 2017; Sargazi et al., 2022). Core-shell NPs is one of the examples that can act as a nanocarrier for therapeutic agents. Core-shell NPs consist of a core and a shell that is coated on top of the core which can be constructed by both organic and inorganic materials to achieve the desired effects and properties (Ghosh Chaudhuri and Paria 2011). Core-shell NPs exhibit unique and superior properties as compared to the individual NPs counterparts due to the bifunctional effects (Liu and Priestley 2016). These unique properties such as biocompatibility, easy surface modifications, and tunable surface plasmonic resonance properties arise from the large surface area of their nano sizes (1–100 nm) and the synergy of the core with the shell material (Matlou and Abrahamse 2022). These characteristics have numerous advantages for therapeutic applications such as the increasing of residence time, bioavailability, dispersity, stability, and the reduction of active dosage (Chatterjee et al., 2014). Fabrication of gold-silver core-shell NPs (Au-AgNPs) is of great interest because they are useful not only as a drug carriers but have also shown distinct optical properties that are beneficial in cancer marker studies and imaging (Gheorghe et al., 2011).

The synthesis of NPs can be achieved through conventional method such as chemical and physical method, but these methods are relatively toxic (Alam et al., 2014). In contrast, the environmental-friendly biosynthesis focuses more on reduction and elimination of the use of hazardous chemical substances (Jahangirian et al., 2017). This method is less toxic and more suitable for biomedical applications. In this study, we synthesized the Au-AgNPs from *Garcinia mangostana* (GM) (mangosteen) crude extract. The ratio of gold nanoparticles (AuNPs) and silver nanoparticles (AgNPs) was then optimised to obtain the optimal size and shape. The Au-AgNPs was loaded with protocatechuic acid (PCA) which is a natural-occurring phenolic acid present in most of the edible medicinal plants (Cai et al., 2004). PCA was chosen as it has been previously reported to kill cancer cells by inducing apoptosis (Barahuie et al., 2013). PCA served as a preventive action towards cancers by its antioxidant properties. Generation of free radicals is inhibited by PCA. They also scavenge and increase the catalytic activity of endogenous enzymes involved in the neutralization of free radicals (Tanaka, Tanaka, and Tanaka 2011). However, its anticancer activity is greatly limited by the target selectivity, thermal stability, dosage and timing between administration (Barahuie et al., 2015; Usman et al., 2018a), which can be potentially solved by introducing a nanocarrier. The novelty of this study is the synthesis of core-shell NPs using GM extract as a targeted drug carrier for plant based PCA that highly dependent on the dose used and the timing between administration of the acid. To the best of our knowledge, this is the first report showing the synthesis of core-shell NPs using GM extract that can be used as a drug nanocarrier against human colon cancer cells.

## Materials and methods

### Materials and reagents

GM fruit were collected from an orchard located at Terengganu, Malaysia. Tetrachloroauric (III) acid (HAuCl_4_) (≥99.9%) and silver nitrate (AgNO_3_) (>99.8%) which act as precursors were purchased from Sigma-Aldrich and Acros Organics, respectively. Phosphate buffer saline (PBS) solution was purchased from R&M Chemicals. Protocatechuic acid (PCA) (≥97%) was purchased from Sigma-Aldrich. All reagents used were of analytical grade.

### Preparation of *G. mangostana* fruit peels extract

GM peels were collected and washed thoroughly with tap water to remove dirt. The peels were washed again with distilled water before drying in an oven at 40°C. All the peels were ground into fine powder using an electrical blender and stored in an air-tight bottle at room temperature for future use. When needed, fruit peels extract was prepared using fine powder of GM (0.5 g) added into 20 ml of deionised water and stirred at 60°C for 30 min. Crude peels extract was filtered and used for the synthesis of NPs.

### Synthesis of gold nanoparticles

AuNPs was synthesized based on our previous work with slight modification in which the synthesis parameters were optimised to obtain AuNPs so that the size and shape is suitable to act as the core of Au-AgNPs (Lee et al., 2016; Lee et al., 2017). AuNPs were used as the core NPs for the formation of Au-AgNPs which is described below. 2 ml of 10 mM HAuCl_4_ was mixed with 20 ml of GM crude peels extract that was heated at 45°C with pH 4. Colour change of the reaction was observed and recorded. AuNPs was characterised as shown in Supplementary Data A.

### Synthesis of gold-silver core-shell nanoparticles

Au-AgNPs were synthesized through seed-mediated method adapted by Wang et al. (2016) with slight modification. A series of mixture (10 ml) was prepared by mixing different ratio of the AuNPs seeds solution with 7.5 mM AgNO_3_ aqueous solution. After that, 10 ml of 25 mg/ml GM crude extract was added immediately into the mixture. The reaction system was stirred continuously with 500 rpm for 5 h. Temperature of the reaction system was kept constant at 45°C in a water bath. The colour change of solution was observed and UV-vis analysis was carried out instantly after the reaction.

### Loading of protocatechuic acid into gold nanoparticles and gold-silver core-shell nanoparticles

10 ml of Au-AgNPs and AuNPs suspensions were added into 50 ml of 5 mg/ml PCA aqueous solution. The reaction was mixed under ultra-sonication (30 amplitudes) for 1 h with 1 s on and off to improve the interaction between PCA and NPs. The obtained drug-loaded NPs (AuPCA and Au-AgPCA) were centrifuged and washed repeatedly three times before drying in an oven for further characterization.

### Characterisation of gold nanoparticles and gold-silver core-shell nanoparticles

The formation of AuNPs and Au-AgNPs were determined by UV-visible Spectrometer (Shimadzu UV-2600) in the range of 300–800 nm. Morphology, size and distribution of the samples were captured by high-resolution transmission electron microscope (HR-TEM, JEM-2100F). Surface morphology of the NPs was observed using field emission scanning microscope (JEOL-FESEM, model JEM-2100F) at accelerating voltage of 15 kV and working distance of 21–22 nm. EDX system (OXFORD) with XMAX 50 detector was used to obtain the EDX result. Elemental composition of the samples was measured using AXIS-ULTRA (Kratos Analytical). Functional groups present in the synthesized NPs were determined using Fourier transform infrared spectroscope (Shimadzu IRTracer-100). NETZSCH STA 449F3 STA449F3A-1108-M was used to conduct thermogravimetric analysis (TGA) to determine the loading of drug on the NPs. Zeta potential and hydrodynamic radius of the samples were recorded using Anton Paar Litesizer 500. 10 drops of all the samples were diluted with 3 ml of PBS (pH 7.4) before running the zeta potential test.

### Drug release

5 mg PCA-loaded NPs were suspended in 5 ml PBS adjusted to pH 5.0 (mimicking the intratumoural acidic pH) placed into dialysis bags with the two ends tied) (Walker and Walker 1992; Jain 2017). The dialysis bags were immersed fully into 40 ml of PBS with constant stirring of 100 rpm. The whole system was incubated at 37°C. 1 ml aliquot was sampled from the system at chosen time intervals (0, 15, 30, 45 min, 1, 2, 3, 4, 6, 8, 10, 24, and 26 h) for analysis. After the removal of aliquot, an equal volume of fresh medium was immediately replenished to keep the volume constant. The collected samples were then analysed using UV-visible spectrometer at 250 nm. All of the experiments were repeated in triplicate.

### Cell culture

HCT116 (ATCC CCL-247) colorectal carcinoma and CCD112 (ATCC CRL-1541) colon normal cell lines were purchased from American Type Culture Collection (ATCC, United States). Both cell lines were maintained in Dulbecco’s Modified Eagle’s medium (DMEM) supplemented with 10% fetal bovine serum (FBS) (Gibco) and 1% penicillin/streptomycin (Gibco). CellTiter 96 Aqueous One Solution or MTS reagent (#G3582, Promega) was purchased to evaluate the cytotoxicity of AuNPs, Au-AgNPs and Au-AgPCA NPs on the cell lines as described below.

### Cytotoxicity assay

To determine the cellular killing effect of AuNPs, Au-AgNPs, and Au-AgPCA NPs, MTS assay (Promega) was performed according to the manufacturer’s instruction with slight modification as previously described by our group (Sukri et al., 2019). Briefly, 5,000 HCT116 and CCD112 cells per well (100 µl/well) were seeded onto a 96-well plate and incubated overnight at 37°C in a 5% CO_2_ incubator. The next day, 2-fold serially diluted NPs (250, 125, 62.5, 31.3, 15.6, 7.8, 3.9, 0 μg/ml) (100 µl/well) were added into the wells and the plate was incubated for 72 h at 37°C in the 5% CO_2_ incubator. Then 20 µl MTS reagent per well was added into the plate and incubated for additional 3 h at 37°C in the 5% CO_2_ incubator. The optical density (OD) was then measured at 490 nm using a multimode microplate reader (Tecan). The dose-response graph was plotted by calculating the percent cell viability using the formula below:

$$\%\;Cell\;viability=\frac{mean\;\left({OD}_{sample}-{OD}_{blank}\right)}{mean\;\left({OD}_{control}-{OD}_{blank}\right)}\times 100\;$$ (1)

In addition, the inhibitory concentration causing 50% growth inhibition (IC_50_ value) was also determined using an online calculator (https://www.aatbio.com/tools/ic50-calculator).

### Live/dead cell staining

Live/dead cell staining assay was performed as described previously with some modifications (Yusefi et al., 2021). HCT116 and CCD112 cells were seeded and treated with 15 μg/ml PCA, Au-AgNPs, and Au-AgPCA, respectively as described above. After 72 h, the live/dead cell status of treated cells were stained with 0.5 μM Calcein AM (#C1430, Invitrogen) and 50 μg/ml propidium iodide (PI) (#556463, BD Pharmingen) for 1 h at 37°C before viewing under the inverted fluorescence microscope (Nikon Eclipse Ti-S, Japan). Calcein staining was observed using green fluorescence filter (excitation maximum 388 nm, emission maximum 530 nm) while PI staining was observed using red fluorescence filter (excitation maximum 535 nm, emission maximum 617 nm). Images were captured at different magnification and overlaid using the Nikon NIS-Elements (Japan) microscope imaging software.

### Statistical analysis

Independent experiments were repeated at least three times, and the data were expressed as mean ± standard deviation for all triplicates within an individual experiment. Data were analysed using a Student’s *t*-test. *p* < 0.05 was considered significant.

## Results and discussion

### Synthesis and characterisation of gold-silver core-shell nanoparticles

**FIGURE 1:**
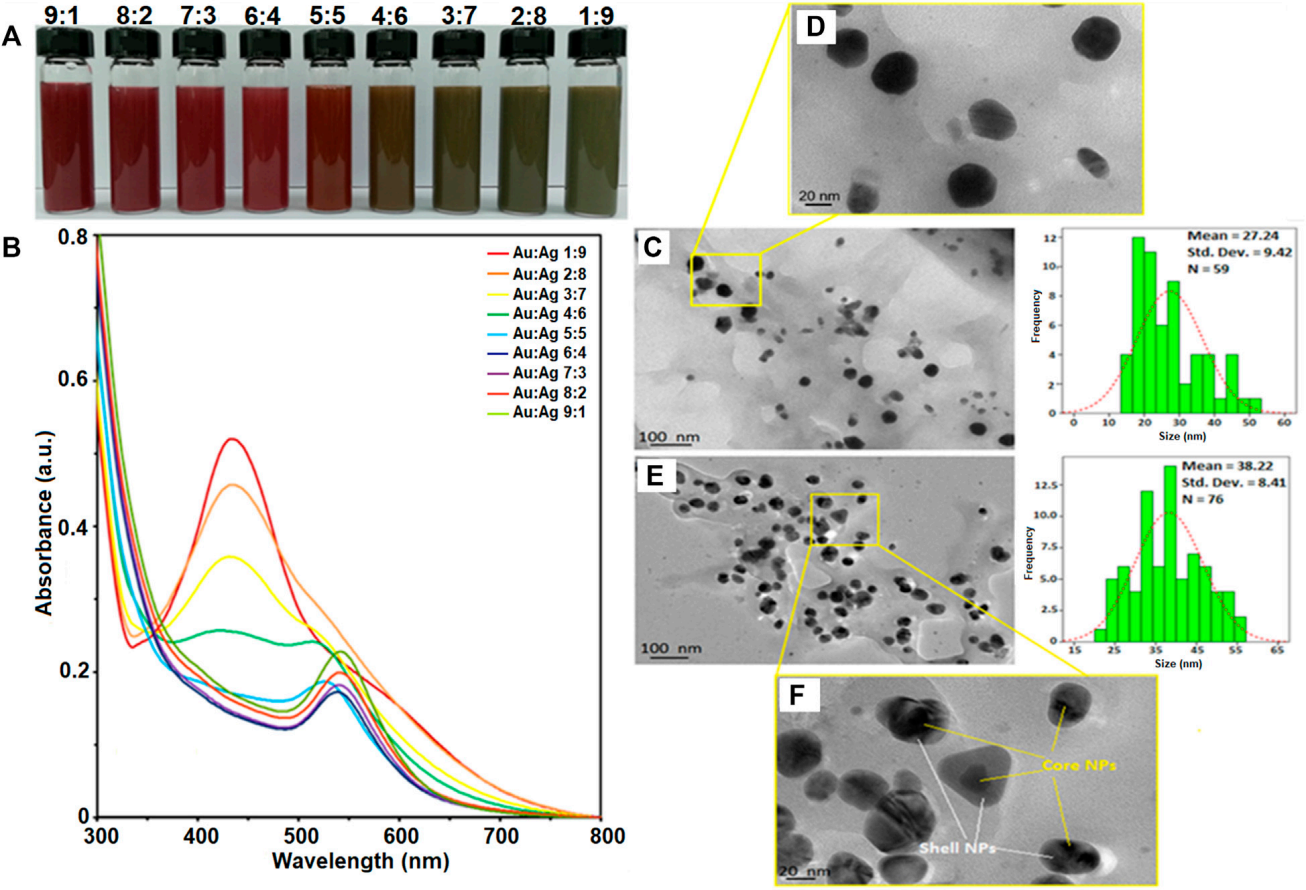
Characterisation of gold-silver core-shell nanoparticles (Au-AgNPs). **(A)** Colour changes of reaction, **(B)** UV-vis spectra of the sample at different ratios of Au:Ag, **(C)** Size and shape of sample Au:Ag 9:1 by TEM image, **(D)** Zoomed in TEM image of sample Au:Ag 9:1, **(E)** Size and shape of sample Au:Ag 1:9 by TEM image and **(F)** Zoomed in TEM image of sample Au:Ag 1:9.

Gold-silver core-shell nanoparticles (Au-AgNPs) was synthesized *via* seed germination method. AuNPs seed synthesised was discuss in our first paper where AuNPs was synthesized using GM peel extract through bottom up synthesis. In this study, different concentration of GM peel extract was reacted with HAuCl_4_ to form circular NPs (Lee et al., 2017; Lee et al., 2019). The synthesis method was optimised to produce AuNPs nano-seed as shown in Supplementary Data A (Baran et al., 2021a). Au nanoseed was synthesized firstly and mixed with silver nitrate (AgNO_3_) to form core-shell NPs. Figure 1 shows the colour changes in the reaction, UV-vis spectra of Au-AgNPs at different ratios, and TEM images of Au:Ag 1:9 and Au:Ag 9:1 samples. Colour changes were observed when different ratios of AuNPs and AgNO_3_ reacted as shown in Figure 1A. The colour changes depended on the amount of AgNO_3_ solution, wherein the colour changed from wine red to brown, and then to greyish brown following the increased amount of AgNO_3_. Ag^+^ ions dissociated and reacted with AuNPs, fresh GM extract was then added to reduce the Ag^+^ into Ag^0^. A layer of Ag shell was absorbed onto the Au seeds and grew epitaxially (Wang et al., 2016).

Au-AgNPs solution was generated and analysed by UV-visible spectroscopy to confirm the formation of core-shell NPs. Figure 1B shows the UV-vis spectra of Au-AgNPs synthesized at different ratios of Au:Ag. At the ratio of 9:1 (Au:Ag), a sharp peak at 544 nm was observed. This peak corresponds to AuNPs peak which is in accordance with the AuNPs previously synthesized by our group (Lee et al., 2017). The complete characterization data of AuNPs is shown in Supplementary Data A. At the ratio of 6:4 (Au:Ag), the intensity of peak dropped and the absorption band broadened. The increased peak intensity suggested the increase of particle size or possible aggregation (Wang et al., 2016). At ratio 4:6, 3:7, and 2:8, the UV-vis spectra showed two plasmon bands: one broader plasmon band at 430 nm and another weak shoulder band at 519 nm, indicated the initiation of Au_core_/Ag_shell_ NPs formation which is consistent with previous finding (Zhang et al., 2013). A peak at 430 nm is corresponded with AgNPs while 519 nm is corresponded with AuNPs (Lee et al., 2017; Baran et al., 2021b), and started to form as shown for sample 3:7 and 2:8 (Figure 1B). The difference between UV profile of 4:6 and 3:7 suggested that pure silver critical shell thickness was exceeded and hence, a peak started to form at 400–500 nm (Zhang, Okuni, and Toshima 2011). As the ratio of Ag increased to 1:9, a sharp plasmon band at 434 nm was recorded and the Au plasmon at 544 nm disappeared. When AgNPs was successfully coated around AuNPs seed, the surface plasmon resonance (SPR) band of AuNPs was sealed by the AgNPs. Hence, UV-vis spectrophotometer could only detect the SPR signal of AgNPs (Zhang et al., 2013). Therefore, this result of sample 1:9 suggested that Ag was successfully coated around Au seed, assuming that the coating was continuous and uniform. The increased peak intensity of Ag for sample 1:9 suggested that the particles were well-dispersed through the shell deposition process and the NPs formed corresponded to the Ag characteristics (Lu et al., 2013; Berahim et al., 2018). Therefore, sample Au:Ag 1:9 was chosen for further analysis of the purity, shape and size.

Figure 1C shows the sample of Au:Ag 9:1 where most of the NPs were spherical in shape with some irregular and rod shape. The average size of the Au-AgNPs was 27.24 nm, which was bigger than the AuNPs (17.95 nm in size after optimisation). Even smaller NPs (10–15 nm) were also observed which could consist of the AgNPs that were not fully coated on the surface of Au seed. When the ratio of Ag increased to 9, the size of Au-AgNPs increased to 38.22 nm as shown in Figure 1E. The distribution and size of the NPs was more uniform for sample Au:Ag 1:9 as compared to 9:1. This result was in line with the previous report which demonstrated that Au-AgNPs formed spontaneously *via* self-organisation (Zhang et al., 2013; Prabhawathi et al., 2019). Well-separated core-shell NPs were observed in Figure 1F. NPs were properly capped in which a darker spot was observed in the centre while a lighter shell was observed surrounding the darker spot. The observation on Au:Ag 1:9 was similar to a previous study showing the formation of core-shell NPs (Karthika et al., 2017). These findings showed that GM extract can act as a reducing agent for the synthesis of core-shell Au-AgNPs with desired particle size as compared to previous studies in which chemical method were adopted (Gheorghe et al., 2011; Lu et al., 2013; Tripathy et al., 2017).

**FIGURE 2:**
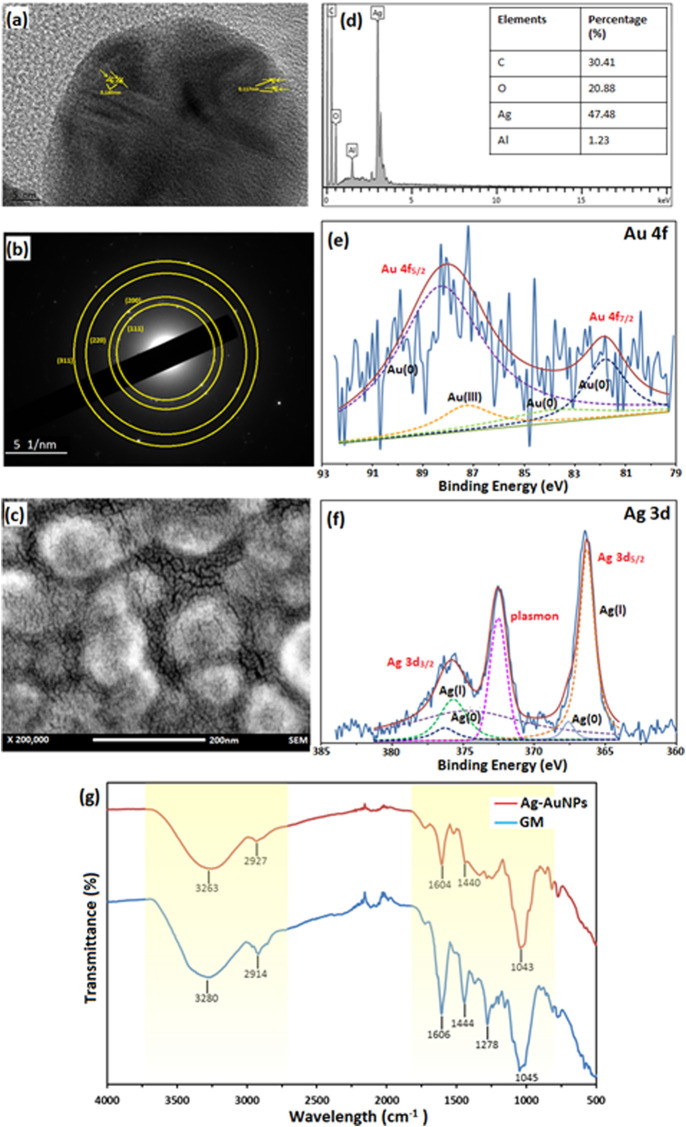
Characterisation of sample Au:Ag 1:9. **(A)** Lattice spacing of TEM image, **(B)** SAED of Au-AgNPs, **(C)** FESEM image of Au-AgNPs, **(D)** EDX spectra, **(E)** XPS spectra of Au-AgNPs (Au 4f band), **(F)** XPS spectra of Au-AgNPs (Ag 3d band), and **(G)** FTIR spectrum of Au-AgNPs and GM extract.

The sample Au:Ag 1:9 was further analysed to determine the physicochemical properties of core-shell NPs as shown in Figure 2. Lattice spacing of the sample Au:Ag 1:9 resulted in the measurement of 1.40 Å and 1.17 Å, respectively as shown in Figure 2A. The measurement corresponded to plane (220) and (222) of fcc structure, respectively. The metallic property of Au-AgNPs was further supported by SAED result in Figure 2B. Ring patterns that can be indexed to (111), (200), (220), and (311) were obtained and the result was in line with the database on JCPS file no. 00-004-0783 and 00-004-0784 (Saikia et al., 2018). Using XRD peak values, a crystal size of 24.55 nm were calculated according to the Debye-Scherrer equations with the formulation of [D = Kλ/(βcosθ)] where D is the particle size, K is the constant (0.9), *λ* is the X-ray wavelength value (1.5418 Å), *ß* is the value of peak at maximum height and *θ* is the Bragg angle of the highest peak (Baran et al., 2021a). The slightly smaller in size is due to the presence of some smaller NPs of Ag that is not filly coated on the AuNPs surface. Since AuNPs and AgNPs are crystallite fcc structure in nature, therefore they have a similar lattice spacing and planes (Sapkota and Han 2017).

From the field emission scanning electron microscopy (FESEM) image in Figure 2C, core-shell NPs were spherical in shape and well-dispersed without agglomeration. EDX analysis recorded 47.48% of Ag on the surface of the NPs which suggested that AgNPs was coated uniformly around the core (Figure 2D). Traces of carbon and oxygen that were recorded on the surface of the Au-AgNPs also suggested that GM matrix acts as the stabilising agent in the reduction process.

In Figures 2E,F, the deconvolution of X-ray photoelectron spectroscopy (XPS) confirmed the presence of Au and Ag through a doublet representing Au 4f_5/2_ and Au 4f_7/2_ in the metallic 4f band of Au with binding energy of 88.1 and 83.74 eV (Sylvestre et al., 2004; Sapkota and Han 2017). Deconvoluted Au 4f spectra consisted of four peaks which corresponds to Au^3+^ (87.3 eV) and Au^0^ (88.3, 83.9, and 81.8 eV) (Sylvestre et al., 2004; Bhandari et al., 2011). Other than that, two peaks that represented Ag 3d band were observed with binding energy of 375.9 eV (Ag 3d_3/2_) and 366.3 eV (Ag 3d_5/2_). Two peaks were attributed to Ag 3d_3/2_ that were Ag+ and Ag^0^ with the binding energy of 375.7 and 374.7 eV respectively (Zhang et al., 2014; Kumar-Krishnan et al., 2015). Another two peaks that attributed to Ag 3d_5/2_ were 366.3 eV for Ag+ and 367.6 eV for Ag^0^ (Shen et al., 2015). This result suggests that most of the Ag exist in ionic state and part of them had been reduced to metallic AgNPs (Kumar-Krishnan et al., 2015). Plasmon was observed at 372.5 eV, it is related to the energy loss when the photoelectron interacted with other electrons.

Lastly, FTIR results in Figure 2G further supported that GM matrix reacts closely in the stabilising process. The major stretching appeared at the region of 3,000–3,500 cm^−1^ and 1,000–1,600 cm^−1^ for both GM peels and Au-AgNPs. Stretching at 3,263 cm^−1^ (Au-AgNPs) and 3,280 cm^−1^ (GM) are related to O-H functional group. A shifting was observed at this region for Au-AgNPs suggested that this group involved in the reduction and capping of the core-shell NPs (Sapkota and Han 2017). At 2,914 cm^−1^ (GM) and 2,927 cm^−1^ (Au-AgNPs) presence of C-H bond in xanthone and other compounds in the peels extract (El-Naggar et al., 2016) were detected. On the other hand, peaks around area 1,606 and 1,444 cm^−1^ for both GM and Au-AgNPs were associated with C=C aromatic ring structure (Wang et al., 2016). Finally, C-O-C stretch can be found in the range of 1,000–1,300 cm^−1^ where the peak at 1,278 cm^−1^ for GM disappeared after the reaction with Au-AgNPs (Sathiyanarayanan et al., 2014). All of the above analysis suggested that GM peel extract reacted closely to form and stabilise the NPs (Shankar et al., 2004).

**FIGURE 3:**
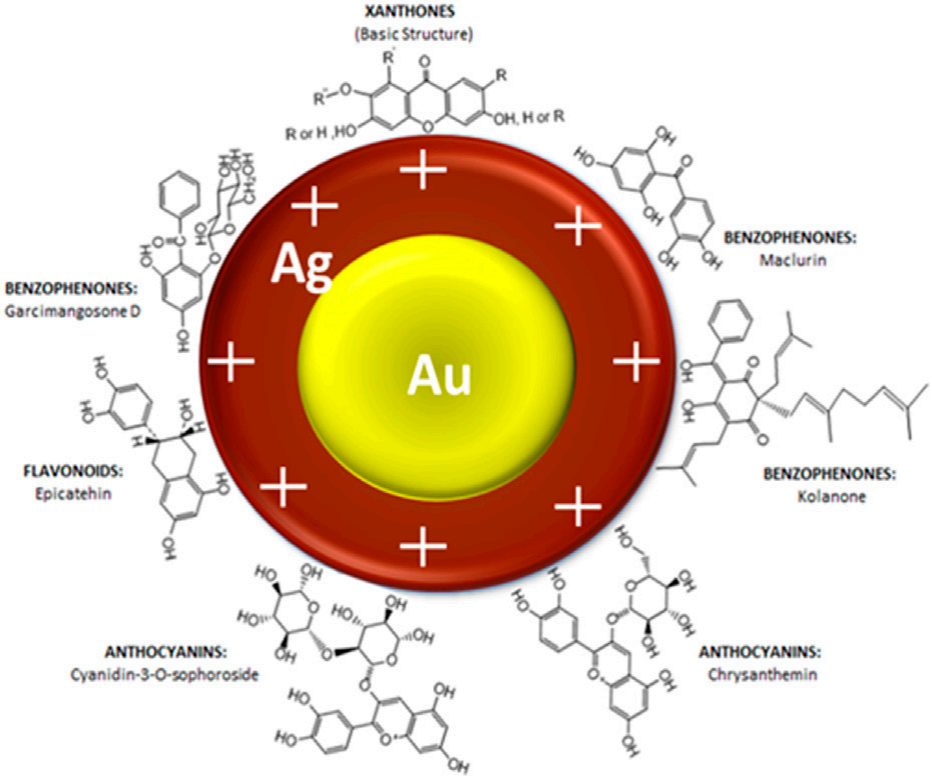
Schematic illustration of interaction between Au-AgNPs and the active functional groups present in GM peels extract.

A schematic illustration of the stabilised Au-AgNPs is shown in Figure 3 based on the known organic compounds present in GM peels extract. FTIR analysis suggested that hydroxyl groups are involved closely in the stabilisation of Au-AgNPs. Surface of the core-shell NPs is speculated to be positively charged in order for hydroxyl groups to stabilise the NPs as supported by the finding from FTIR. Since the surface of Au-AgNPs is speculated to be positively charged, the loading of PCA could took place at the–COOH groups (Barahuie et al., 2015) (Effective cancer treatment by targeted pH sensitive–gold nanoparticles without using laser irradiation).

### Drug loading and drug release study

**FIGURE 4:**
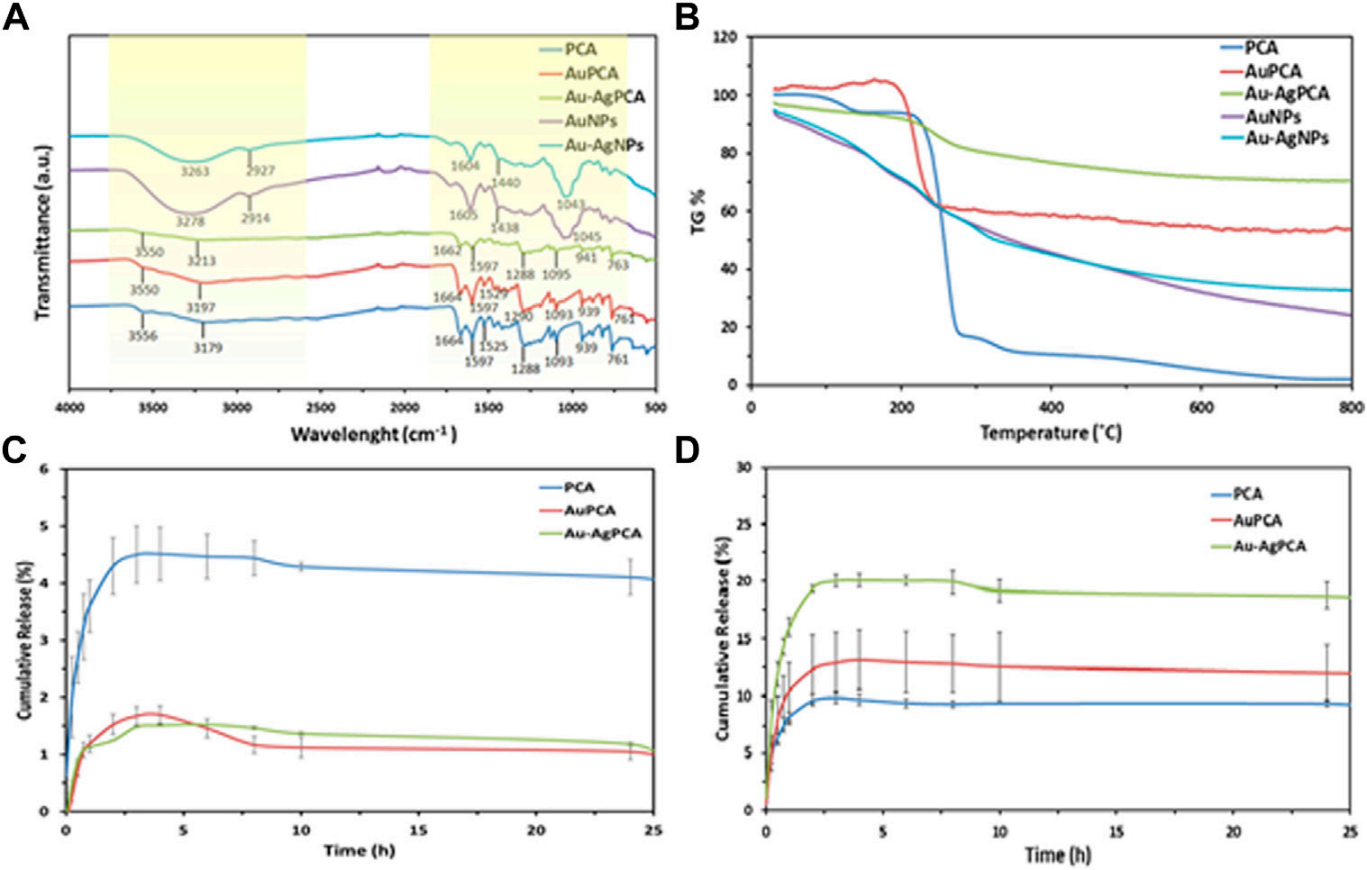
Drug loading of AuPCA and Au-AgPCA based on **(A)** FTIR, **(B)** TGA and **(C)** drug release of AuPCA and Au-AgPCA at pH 7 and **(D)** pH 5.

Protocatechuic acid (PCA) was loaded into the synthesized AuNPs and Au-AgNPs through sonication method to improve the interaction between PCA and surface of the NPs. The successful loading of PCA was shown by UV-vis spectroscopy where two peaks corresponding to PCA were observed at 251 and 288 nm as shown in Supplementary Data B. Peaks at the range of 225–325 nm were highlighted and enlarged because the samples were diluted so that the loading peaks could be recorded. The PCA-loaded NPs were then analysed by FTIR and thermogravimetric analysis (TGA). FTIR spectra of PCA, PCA-loaded AuNPs (AuPCA) and PCA-loaded Au-AgNPs (Au-AgPCA) were studied as shown in Figure 4A to understand the interactions between the functional groups of PCA and the NPs that occurred during the loading process. Clear differences were observed between the spectra of bare NPs and PCA-loaded NPs. The spectrum of PCA-loaded NPs was comparable to PCA spectrum suggesting that the reaction occurred between PCA and NPs. O-H stretch was observed around the region of 3,200 and 3,550 cm^−1^ for all of the three samples. Shifting from 3,179 to 3,213 cm^−1^ occurred for sample Au-AgPCA and the reduced intensity of 3,213 cm^−1^ stretch suggested that hydrogen bonding occurred between the drug and Au-AgNPs nanocarrier (Usman et al., 2018b). The peaks at 1,288 and 1,664 cm^−1^ were attributed to C=O stretching of carboxylic group for all three tested samples (Barahuie et al., 2013). Slight shifting and decrease in intensity also occurred to the 1,288 cm^−1^ band after loading with different NPs. This is due to the occurrence of hydrogen bonding between PCA and the NPs (Usman et al., 2018a). Peaks at the region of 1,525 and 1,597 cm^−1^ for all three samples were attributed to aromatic C=C bands (Xu et al., 2018). 1,093–1,095 cm^−1^ for C=O bond was observed in the FTIR spectra of all samples (Usman et al., 2018b). Similarly, reduced intensity was observed at 941 cm^−1^ for Au-AgPCA. The reduced intensity and shifting at 939 cm^−1^ suggests that the reaction took place between the–COOH groups of PCA and NPs (Barahuie et al., 2015). Finally, peaks at 761–763 cm^−1^ corresponded to C-H bonding of all samples (Saifullah et al., 2018).

Figure 4B demonstrates the thermal analysis of the bare NPs, PCA, and PCA-loaded NPs. Bare NPs showed gradual weight loss due to the gradual combustion of metallic NPs. PCA showed three main weight loss stages. The first weight loss stage (4.64%) corresponded to the loss of absorbed water at 138°C based on a previous study (Barahuie et al., 2015). Second stage of weight loss (77.64%) occurred at 200°C–299°C. This was related to the decomposition combustion of PCA (Barahuie et al., 2013). The last stage of weight loss (6%) occurred at the region of 299°C–350°C which was due to the decomposition of PCA residue (Usman et al., 2018a). The weight loss of PCA ended at 348.7°C. Drug-loaded NPs only showed a single stage of weight loss at the region of 240°C–299°C which was due to the combustion of PCA. The drug-loaded NPs appeared to be more thermally stable as compared to PCA. The TGA result suggested that the stability of PCA increased after being loaded on the nanocarriers.

From the TGA findings, encapsulation efficiency and drug loading efficiency were calculated as shown in Table 1. Mass loss of each of the samples was determined at 348.7°C. AuNPs have a higher loading with PCA (40.67%) mainly due to its multisurface functionality (Ovais et al., 2017). The drug loading capacity of Au-AgNPs was lower (21.26%), this is because AuNPs were fully coated by AgNPs, hence reducing the loading property of the Au-AgNPs.

**TABLE 1:** Mass loss and drug loading capacity of PCA, AuPCA and Au-AgPCA.

Sample	Mass loss at 348.7°C (%)	Drug loading capacity (%)
PCA	88.28	—
AuPCA NPs	40.67	40.67
Au-AgPCA NPs	21.26	21.26

PCA, protocatechuic acid; NPs, nanoparticles; AuPCA, Gold NPs loaded with protocatechuic acid; Au-AgPCA, Gold-silver NPs loaded with protocatechuic acid.

As shown in Figures 4C,D, the release of PCA was observed under neutral condition (pH7.4) and a slightly acidic condition (pH 5.0), which mimics the tumour microenvironment with actively proliferating tumour cells. Based on Figure 4C, the released was completed in about 5 h. For PCA the maximum released was 4.5%, while for AuPCA and Au-AgPCA was 1.7% and 1.5% respectively. Based on Figure 4D, the cumulative release percent of AuPCA at pH 5 was 13%. The release rate of Au-AgPCA was faster as compared to the AuNPs with a cumulative release of 20% in 6 h at pH 5 while AuNPs cumulative release in 6 h is around 13% only. The release of all samples achieved equilibrium after 12 h and no further changes up to 25 h. Sustain release of medication throughout the day (24 h) will reduce the dosage of drug taken. Hence, for nanoparticles drug delivery system, the timing of release is crucial to enable its release at target side continuously instead of dissociate at non-desirable areas (Patra et al., 2018). The cumulative release of the samples at pH 7 is much lower than pH 5, suggesting that the samples could target cancerous cells at specific pH environment (Yew et al., 2019). Ag-AuPCA suggest a passive targeting pathway by direct chemical conjugation where the drug carrier complex circulates in bloodstream and driven to target side by the influence of pH (Lu et al., 2016) Burst release was observed in all samples at the first 15 min as shown in Figure 4D. This phenomena indicates that PCA was weakly bound or adsorbed on the large surface area of the NPs (Villaverde 2011; Barahuie et al., 2015). After that, the remaining PCA were released in a gradual and sustained manner for about 6 h before reaching plateau. The continual release of PCA in Au-AgNPs was due to drug diffusion and surface erosion of the NPs (Duzgunes 2012). This could be explained by the hydrogen bonding between PCA and Au-AgNPs as shown by FTIR earlier (Usman et al., 2018a; Usman et al., 2018b). PCA solution that acts as a control had a rapid release and completed the process in about 3 h with a cumulative release of 9.7% as shown in Figure 4D. Rapid release of PCA occurred because the PCA was dispersed freely in the system.

Table 2 shows the zeta potential and hydrodynamic radius of various NPs. All NPs possessed negatively charged surface which is consistent with the FTIR data that suggested hydroxyl groups took part in the stabilisation of the NPs, forming an anions layer on the NPs surface (AL-Jawad et al., 2019). All of the NPs are considered stable as for a suspension system, zeta potential value of ±20 mV is enough because of the present of combined electrostatic and steric stabilization among the NPs in the system (Jacobs et al., 2002). The zeta potential of AuNPs (−23.7 mV) and AgNPs (−21.4 mV) decreased to −25.8 mV after the formation of Au-AgNPs, suggesting that electrostatic interactions occurred between the two NPs (Zhang et al., 2020). After the loading of PCA, zeta potential of Au-AgPCA NPs increased to −21.3 mV which suggests the successful coating of PCA on Au-AgNPs following the interaction with–COOH groups as shown by the FTIR data (Jiang et al., 2019). The hydrodynamic radius data showed the increased size of Au-AgNPs (275.4 nm) which supports the formation of core-shell. The reduced hydrodynamic radius of PCA-loaded Au-AgNPs (249.5 nm) indicated that aggregation was unlikely to occur after the drug loading (George et al., 2020). The hydrodynamic radius value is higher than the particles size calculated by TEM because of nanoparticles surface hydration (Martínez et al., 2020). TEM image only gave information about the diameter of the core of the nanoparticles (George et al., 2020). In this case, the NPs are not suitable to measure using particle size analyser because the NPs are in solid form that does not disperse well in solvent. The very high value of hydrodynamic radius suggest that the NPs particles dynamically aggregate and de-aggregate, given the large value result (Eaton et al., 2017).

**TABLE 2:** Zeta potential and hydrodynamic radius of NPs.

Sample	Zeta potential (mV)	Hydrodynamic radius (nm)
Au NPs	−23.7	219.8
Ag NPs	−21.4	249.5
Au-Ag NPs	−25.8	275.4
Au-AgPCA NPs	−21.3	249.5

To understand the mechanism and kinetics in the drug delivery, the drug release data was fitted into five mathematical models (zero order, first order, Hixon-Crowell, Higuchi and Korsmeyer-Peppas). Table 3 shows the correlation coefficient of AuPCA and Au-AgPCA fitted into the different models. It was found that the release of AuPCA was well-fitted into the Higuchi method with *R* ^2^ of 0.9628. Au-AgPCA also fitted well to this model with *R* ^2^ of 0.9606. This model suggested that the release of PCA occurred *via* diffusion based on the Fick’s Law (Nivethaa et al., 2015). The release data were then fitted into Korsmeyer-Peppas model to understand more about the dissolution mechanism. The *R* ^2^ for all tested samples were in the range of 0.5< *n* < 1 indicating that the drug were released through both diffusion and erosion controlled (non-fickian) method (Safwat et al., 2018). Dissolution-filling approach occurred as PCA gradually released and diffused from the NPs that carried them (Farooq et al., 2018).

**TABLE 3:** Kinetic study assessment of AuPCA and Ag-AuPCA based on various kinetic mathematical models.

Sample	Correlation coefficient of model (*R* ^2^)				
	Zero order	First order	Hixon-Crowell	Higuchi	Korsmever-Peppas
AuPCA NPs	0.7507	0.7662	0.7603	0.9628	0.9448
Au-AgPCA NPs	0.7338	0.7791	0.7638	0.9606	0.9714

NPs, nanoparticles; AuPCA, Gold NPs loaded with protocatechuic acid; Au-AgPCA, Gold-silver NPs loaded with protocatechuic acid.

### *In vitro* anticancer study

Cytotoxic effect of GM peels extract, PCA and other NPs was determined against colon-derived HCT116 (cancer) and CCD112 (normal) cells up to 250 μg/ml. Table 4 shows the determined IC_50_ values for all tested compounds. AuNPs and Au-AgNPs showed cytotoxic effects against HCT116 cells with IC_50_ of 82.99 and 24.36 μg/ml, respectively. When combined with PCA, the IC_50_ values were further reduced except for AuNPs. This result suggested that combining AgNPs and Au-AgNPs with PCA was beneficial in killing the HCT116 cells. AuPCA below 250 μg/ml did not show any cytotoxic effects against both colon cell lines.

**TABLE 4:** IC_50_ values of mangosteen extract, protocatechuic acid, and nanoparticles against two cell lines.

Cell line	IC_50_ (µg/ml)					
	GM extract	PCA	Au NPs	Au PCA NPs	Au-AgNPs	Au-AgPCA NPs
HCT116 (cancer)	35.74	148.09	82.99	>250	24.36	10.78
CCD112 (normal)	49.58	224.39	>250	>250	27.48	43.48

GM, *Garcinia mangostana*; IC, inhibitory concentration; PCA, protocatechuic acid; NPs, nanoparticles; AuNPs, Gold NPs; AuPCA NPs, Gold NPs loaded with protocatechuic acid; Au-AgNPs, Gold-silver NPs; Au-AgPCA NPs, Gold-silver NPs loaded with protocatechuic acid.

**FIGURE 5:**
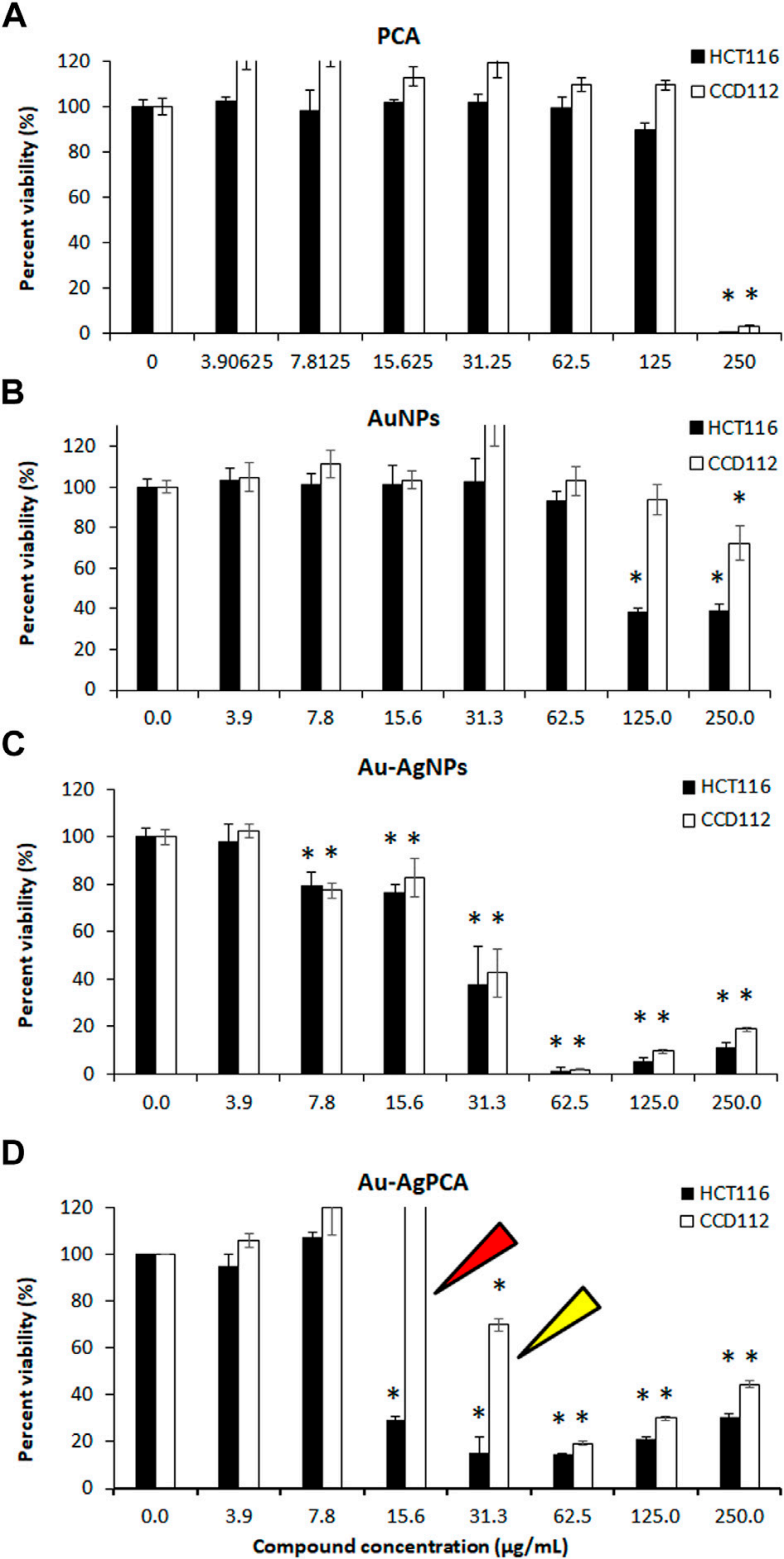
Cytotoxic effects of nanoparticles against colorectal cancer (HCT116) and colon normal (CCD112) cell lines. **(A)** protocatechuic acid (PCA); **(B)** AuNPs; **(C)** Au-AgNPs and **(D)** Au-AgPCA. Arrows show specific cytotoxicity of Au-AgPCA on cancer cells but not in normal cells. Data are expressed as mean ± standard deviation for triplicates within an individual experiment. Statistical significance was performed using Student’s *t*-test. (*p < 0.05).

**FIGURE 6:**
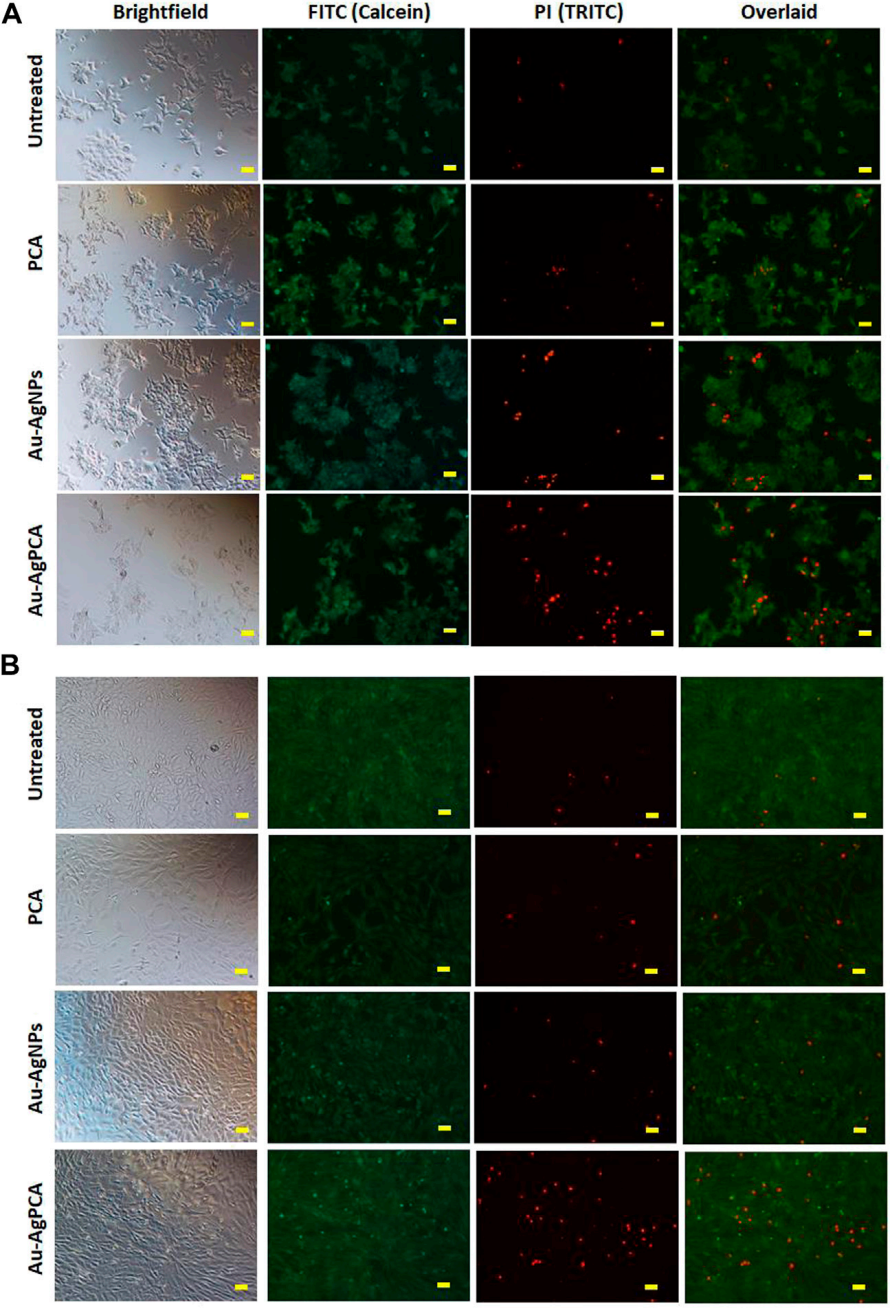
Live/dead cells staining. Calcein AM (green: live cells) and propidium iodide (red: dead cells) staining of **(A)** HCT116 and **(B)** CCD112 cells treated with 15 μg/ml PCA, Au-AgNPs, and Au-AgPCA, respectively. Images were captured at ×10 magnification. Scale bar represents 50 µm.

To examine the selectivity, those NPs which showed lower IC_50_ values in HCT116 compared to CCD112 cells (AuNPs, Au-AgNPs and Au-AgPCA) were selected and replotted as depicted in Figure 5. Overall, dose-dependent inhibitions were observed in the three NPs. However, the level of cytotoxic effect in HCT116 and CCD112 did not show any difference in Au-AgNPs in most of the tested concentrations. As seen in Table 4, AuNPs alone did not kill CCD112 cells up to 250 μg/ml. However the core-shell Au-AgNPs killed both HCT116 and CCD112 cells almost equally. This killing effect might have attributed to the AgNPs as previously described by our group which showed IC_50_ of 40.2 and 47 μg/ml against HCT116 and CCD112 cells, respectively (Lee et al., 2019). Another possible explanation might be the free Ag^+^ ions which have caused such non-specific toxicities. In contrast, AuNPs and Au-AgPCA in some of the tested concentrations showed higher cytotoxic effect in HCT116 compared to CCD112. This result suggests the selectivity of the NPs which is consistent with previous findings (Dykman and Khlebtsov 2012). Interestingly, 15.6 μg/ml Au-AgPCA could kill almost 70% of HCT116 while did not affect the CCD112 cells (shown by a red triangle in Figure 5C). At 31.3 μg/ml, Au-AgPCA showed lower inhibition (30%) towards CCD112 as shown by a yellow triangle. At 15.6 and 31.3 μg/ml, the PCA drug alone did not affect the cell viability of both HCT116 and CCD112 cells. This suggests that Au-AgPCA exibit a better drug specificity against normal cells even at higher dosage and highlights that core-shell NPs has high potential in cancer teatment compared to monometallic NPs (Ghosh Chaudhuri and Paria 2011). Consistent with Figure 5D, live/dead staining of HCT116 (Figure 6A) showed that 15 μg/ml Au-AgPCA killed larger portion of cells (indicated by PI staining and low density of cells in brighfield), but most of the treated CCD112 normal cells remained unharmed as shown in Figure 6B (high density of cells in brightfield). As expected, 15 μg/ml PCA did not affect cell viability of both HCT116 and CCD112 cells which showed similar staining pattern with untreated cells. Meanwhile, 15 μg/ml Au-AgNPs exhibited relatively higher killing in HCT116 cells compared to PCA and untreated, but this was not observed in CCD112 cells.

In short, Au-AgPCA performs better as an anticancer agent against colon cancer cells and meanwhile, it is more specific towards cancerous cells than normal cells. This could be partly attributed to the selectivity of both PCA and AuNPs which showed higher cytoxicity effects against the cancer cells than the normal cells (Table 4). The fact that Au-AgNPs has very similar IC_50_ in both cell lines suggested that the combination of Au and AgNPs was not responsible for the selectivity of Au-AgPCA. Our finding is consistent with a previous study in which doxorubicin-loaded Au-AgNPs was shown to delivered to the HeLa cervical cancer cells and 50% inhibition was observed after 24 h incubation (Chen et al., 2016). As previously reported, doxorubicin-loaded Au-AgNPs could also exhibit a better biocompatiblity and enhanced intracellular binding ability towards the cancerous cells compared to monometallic NPs, hence improving their *in vitro* anticancer action compared to free drug (Jiang et al., 2019). Previous studies have reported that Ag/Au bimetallic NPs could kill HCT116 cells by inducing apoptosis (Katifelis et al., 2022). The triggered apoptosis in HCT116 cells may also be contributed by Ag and Au ions from Ag NPs (Satapathy et al., 2013; Sufyani et al., 2019) and Au NPs (Maddah et al., 2022), respectively. Similarly, apoptosis induction of Ag-NPs was also seen in another colorectal cancer cell line, HT-29 cells (Bao et al., 2022). Besides that, the apoptosis may be also caused by the loaded PCA as previous reports have shown that PCA could trigger apoptosis in another colon cancer cells, CaCo-2 cells (Acquaviva et al., 2021) as well as other cells including breast, lung, liver, cervix and prostate cancer cells (Yin et al., 2009). Other possible pathways that might lead to the cancer cell killing include lactate dehydrogenase leakage (LDH), reactive oxygen species (ROS) generation, impairment of mitochondrial function and DNA damage (Gurunathan et al., 2018).

## Conclusion

This study highlights the green synthesis of Au-AgNPs using *G. mangostana* fruit peel extract and its potential use as a non-toxic nanocarrier for drug delivery. Uniformly distributed spherical shaped Au-AgNPs that is 38 nm was formed when the ratio of Au:Ag was 1:9. The success loading of PCA on the nanoparticles was due to the interaction between–COOH groups of PCA and the nanoparticles. Then, PCA was released from the nanoparticles through both diffusion and erosion controlled based on mathematical models. 15.6 μg/ml Au-AgPCA showed potent inhibition (70%) against colon cancer cell line, HCT116 and remained non-toxic to colon normal cells, CCD112. This highlights the high cancer cell selectivity of Au-AgPCA. At same concentration, both HCT116 and CCD112 did not respond to PCA. Therefore, core-shell Au-AgNPs appear to be an ideal nanocarrier compared to AuNPs and PCA alone in terms of anticancer activity and toxicity.

## Data Availability

The original contributions presented in the study are included in the article/Supplementary Material, further inquiries can be directed to the corresponding authors.
