# Supplementary material for: Potential use of gold-silver core-shell nanoparticles derived from Garcinia mangostana peel for anticancer compound, protocatechuic acid delivery
Source: Front Mol Biosci. 2022 Oct 11;9:997471. doi: 10.3389/fmolb.2022.997471 (PMC9593088; doi:10.3389/fmolb.2022.997471)

Supplementary Data A

UV-vis spectrum of drug (PCA) loaded nanoparticles.

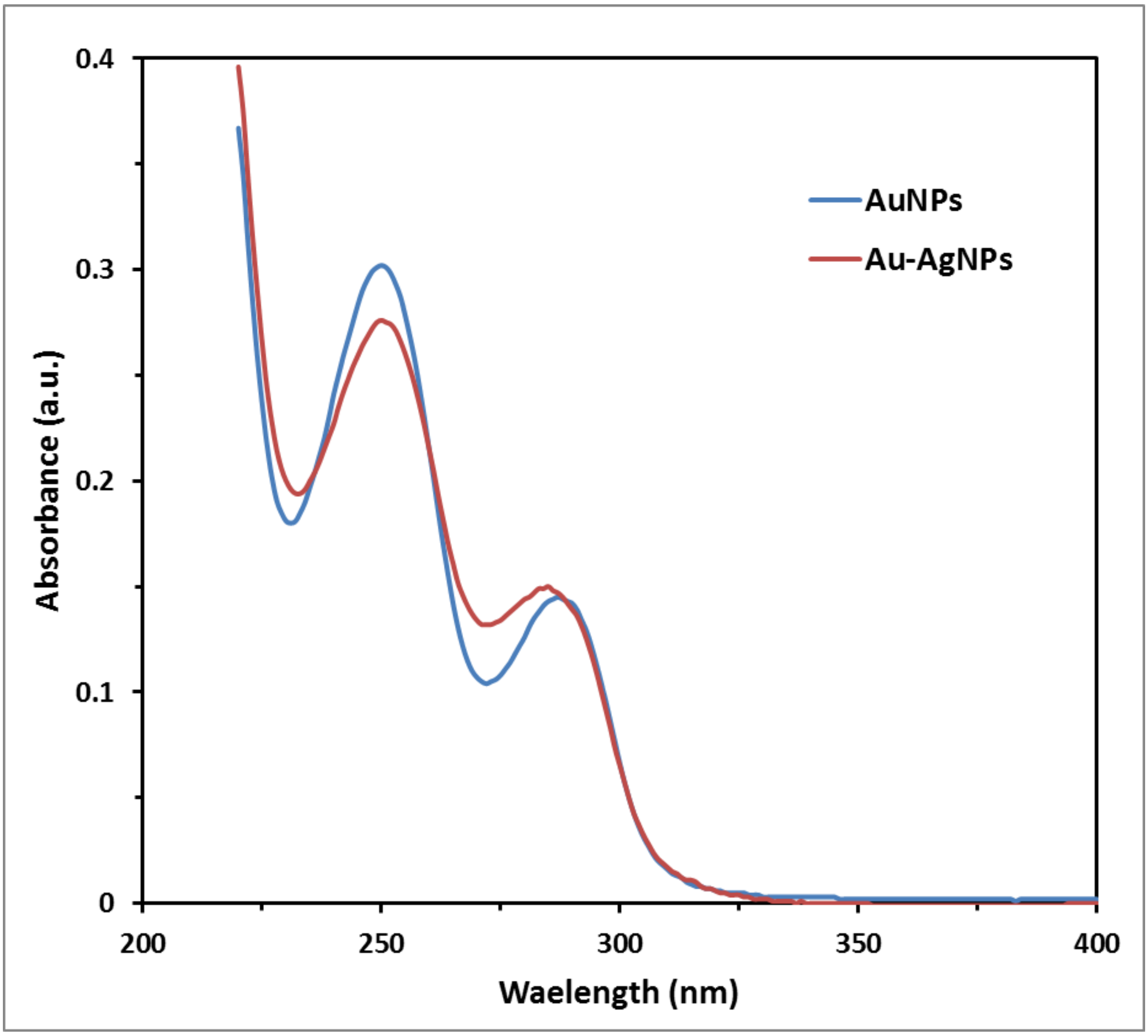

Supplementary Data B

Microscopic analysis of (A) HCT116 and (B) CCD112 cells treated with various concentrations of Au-AgPCA.

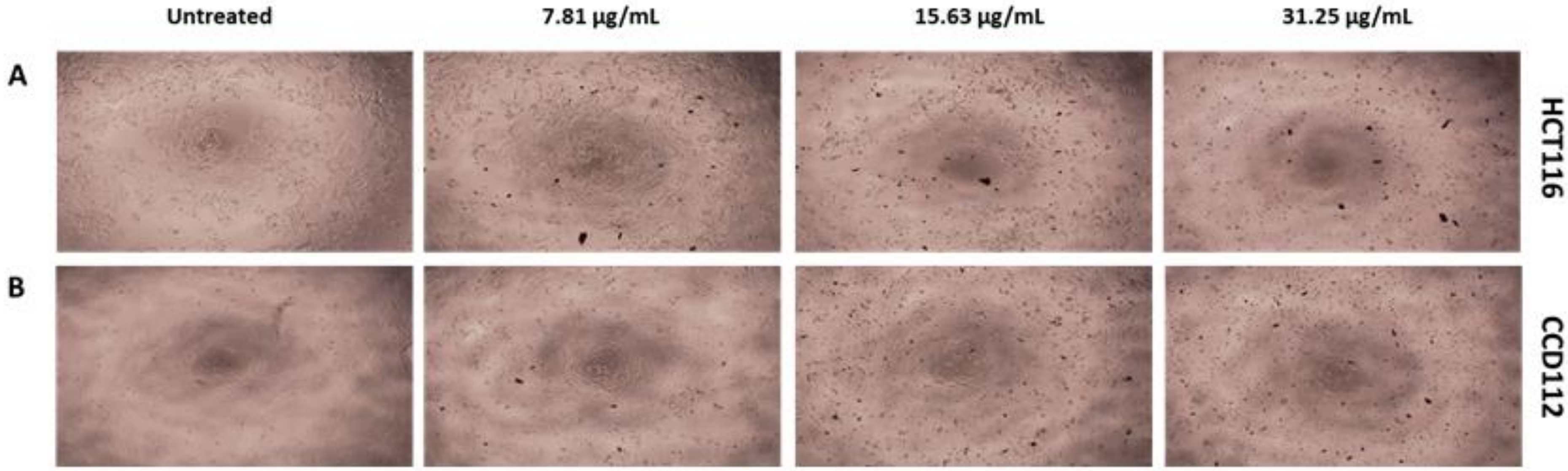

Supplement: Supplementary file 1 [file Presentation1.pdf]
